# Early Activation of Lung CD8^+^ T Cells After Immunization with Live *Plasmodium berghei* Malaria Sporozoites

**DOI:** 10.20411/pai.v10i2.794

**Published:** 2025-03-04

**Authors:** Roos van Schuijlenburg, Chanel M. Naar, Stefanie van der Wees, Severine C. Chevalley-Maurel, Nikolas Duszenko, Helena M. de Bes-Roeleveld, Eva Iliopoulou, Emma L. Houlder, Fiona J.A. Geurten, Els Baalbergen, Meta Roestenberg, Blandine Franke-Fayard

**Affiliations:** 1 Leiden University Centre for Infectious Diseases (LU-CID), Leiden University Medical Centre, The Netherlands

**Keywords:** CD8^+^ T-cells, Lung, LA-GAP, Malaria, Priming

## Abstract

**Background:**

Two novel malaria vaccines, RTS,S and R21, mark a significant step forward in malaria research, but eradication demands vaccines with higher efficacy. Recent trials using late-arresting genetically attenuated parasites (LA-GAP) highlight their effectiveness as next-generation vaccines, likely through CD8^+^ T-cell activation targeting late liver-stage parasites. However, the distribution of LA-GAP-activated T cells in different organs that culminate towards high-level protection in the liver remains unclear.

**Methods:**

This study aimed to map immune responses in the livers and lungs of mice immunized with LA-GAP, shedding light on the role of different organs in priming T-cell responses towards immunity.

**Results:**

Particularly in the lungs we found an impressive increase of CD8^+^, double negative T cells (5%), γδ (2.5%), effector memory CD8^+^ T cells (46%), and tissue resident memory CD8^+^ T cells (3%). These lung T cells are highly activated (expressing CD11c, Ki67, KLRG1) and exhibited 4-fold higher Granzyme A expression and significant TNF^+^ cell increases as compared to their liver counterparts (10.2% vs 2.6%). These differences start already at the early 2-day timepoint at which time the lungs show an impressive 10.2% increase in TNF^+^ CD8^+^ T cells, whereas the liver shows a more modest increase of 2.6% of these cells.

**Conclusion:**

These findings highlight the lungs as a crucial site for immune priming and T-cell activation, underscoring the need for further investigation of organ-specific responses to fully understand the potential of LA-GAP immunization as a powerful strategy in the fight against malaria.

## INTRODUCTION

The rollout of 2 novel malaria vaccines (RTS,S and R21) in sub-Saharan Africa in 2024 for children in medium and high-transmission areas marks the success of decades of malaria vaccine research [1]. These circumsporozoite protein (CSP)-based vaccines now provide the much-needed additional tool to control the increasing number of malaria cases. However, to break malaria transmission, vaccines with higher and broader efficacy are urgently needed. Whole sporozoite (SPZ) vaccination approaches can provide such high protective efficacy, as they allow for the induction of cellular immune responses in addition to CSP-targeting antibodies [2, 3]. Whereas antibodies mediate inhibition of *Plasmodium falciparum* (*Pf*) SPZ motility and invasion of the host, cellular mechanisms are thought to be responsible for killing of the ensuing *Pf*-infected liver cells [4, 5]. This is further supported by our recent finding in an experimental medicine trial where we compared the protective efficacy of immunization with early-arresting (24 hours) genetically attenuated whole SPZ with late-arresting (6–7 days) counterparts in healthy malaria-naïve adults. We found that only the late-arresting genetically attenuated parasites (LA-GAP) were able to efficiently induce protective immunity as tested by controlled human malaria infection [6, 7]. These data, combined with the knowledge acquired from animal models that shows protection against malaria relies on CD8^+^ T cells detecting malaria-infected hepatocytes [8–10], suggest that the activation of CD8^+^ T cells targeting the late liver stage parasite is a critical mechanism of these highly potent whole SPZ vaccines.

Despite multiple human clinical trials with whole SPZ vaccination approaches, very little is known about T-cell mediated activation and killing of liver stage parasites. Signals of polyfunctional CD4^+^ T-cell activation and particularly γδ T-cell activation in peripheral mononuclear cells of protected individuals are not very informative of the mechanism of protection, which is presumed to be located specifically in the liver [11, 12]. Given the inaccessibility of the liver in healthy volunteers, very little is known about priming and potentially boosting of liver-stage specific T cells.

However, in animal models, CD8^+^ T-cell responses after malaria infection have been described in blood, skin, draining lymph nodes, spleen, and liver [13, 14]. The types of immune responses per organ have been found to vary in terms of magnitude and type of responses. For example, skin-based responses are found to be more regulatory with CD8^+^ T-cell priming occurring mainly through dermal CD11c^+^ dendritic cells (DCs) in the skin draining lymph nodes [15, 16]. Splenic DCs were also found to prime CD8^+^ T cells when they encounter SPZ, but the magnitude and durability of the CD8^+^ T-cell response may be influenced by the presence of activated CD4^+^ T cells and γδ T cells [17, 18]. Whereas CD8^+^ T cells are generally thought to circulate, it is the proportion that becomes tissue-resident memory T cells (Trms) that is thought to be the front line of defense for long-lived immunity induced after whole SPZ immunization [19]. A better understanding of the potential priming and boosting of T-cell responses and the ultimate settling of Trms in different organs is critical to better target the LA-GAP immunogen to the appropriate organs and more efficiently induce protection. As SPZ vaccines are currently injected by direct venous inoculation, we argued that it would be critical to follow the immune activation on the migratory path of SPZ in the blood circulation, traveling through the lungs first before reaching their liver target organ.

**Figure 1.**
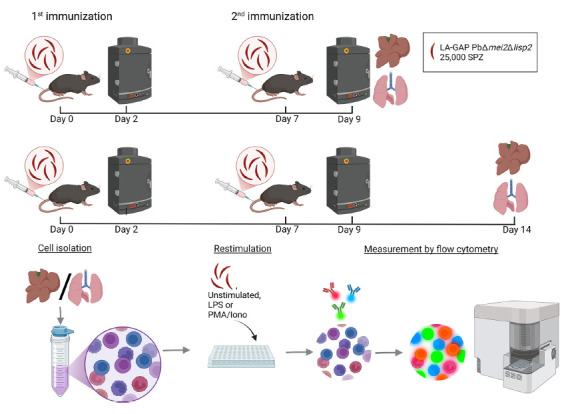
**Experimental setup.** Timeline of the study protocol. Mice were intravenously immunized with 25,000 *Plasmodium berghei* late-arresting genetically attenuated parasites (LA-GAP) PbΔ*mei2Δlisp2*, salivary gland extract (SGE), or medium as negative control in C57BL/6J mice, after which liver load was determined, and organs were harvested 2 days post second immunization or 7 days post second immunization. Created with BioRender.com.

Interestingly, the role of the lung immune system in LA-GAP immunization has been neglected so far, despite the fact that SPZs, when injected into the blood circulation, must recirculate possibly multiple times through the lungs before arriving at the liver. We thus set out to map the immune responses following SPZ immunization in both lungs and livers of mice immunized with LA-GAP (Figure 1).

## METHODS

### Mosquito Production

Mosquitoes from a colony of *Anopheles stephensi* (line Nijmegen SDA500) were used to obtain SPZs. Larval stages were reared in water trays at a temperature of 28 ± 1°C and a relative humidity of 80%. Adult females were transferred to incubators with a temperature of 26 ± 0.2°C and a relative humidity of 80%. For all the experiments, 3- to 5-day old mosquitoes were used. The *Plasmodium berghei* (Pb)-infected mosquitoes were maintained at 21°C at 80% relative humidity.

### Experimental Animals, *P. berghei* Parasite Line

Female OF1 mice and female and male C57BL/6J mice (Charles River Laboratories) of 4 to 6 weeks old were acclimatized for 1 week prior to the experiment. Mice were housed between 4 to 5 mice per cage in ventilated cages with autoclaved aspen woodchip, fun tunnel, wood chew block and nestlets (12:12 hour light-dark cycle; 21 ± 2°C; relative humidity of 55 ± 10%). During the experiment, mice were fed with commercially prepared autoclaved dry rodent diet pellets and water, both available *ad libitum*. All animal experiments were granted with license 11600202216547 by the Competent Authority after advice on ethical evaluation by the Animal Experiments Committee Leiden and were performed in accordance with the Experiments on Animals Act (Wod, 2014), the applicable legislation in the Netherlands in accordance with the European guidelines (EU directive no 2010/63/EU). The study was executed in a licensed establishment for experimental animals.

Mice were euthanized (cardiac puncture under anesthesia) at a parasitemia of 2% to 5% before malaria-associated symptoms occurred. Humane endpoints: the animals' body condition was thoroughly examined daily. Animals were humanely sacrificed if the following defined end points were reached: visible pain (abnormal posture and/or movement), abnormal behavior (isolation, abnormal reaction to stimuli, no food and water intake). No mice were euthanized due to humane endpoints, only one mouse had been excluded due to parasite break-through (explained below).

The LA-GAP PbΔ*mei2*Δ*lisp2* parasite (2900cl3, mutant RMgm-4937; www.pberghei.eu), which is genetically attenuated by the deletion of the meiosis inhibited 2 (*mei2*) and liver-specific protein 2 (*lisp2*) genes [6] was used. Feeding of *Anopheles stephensi* mosquitoes was performed as described previously [20].

### *In Vivo* Immunization of Mice with Pb SPZ

The study including data acquisition was performed blinded. The study was unblinded after data analysis. One day prior to immunizations, mice were randomized into 3 or 4 different groups (4–5 mice per group, maximum of 20 mice per experiment). On the day of immunizations, salivary glands (21–23 days post blood meal) of *P. berghei* LA-GAP PbΔ*mei2*Δ*lisp2* (*Pb* SPZ)-infected mosquitoes were dissected in cold RPMI 1640 glutamax (Thermo Fisher). As a control, salivary glands from the same batch of uninfected mosquitoes, hence referred to as salivary gland extract (SGE), were dissected in cold RPMI 1640 glutamax. Immediately after dissection the glands were crushed and homogenized and the total number of *Pb* SPZ was counted. Dead *Pb* SPZs were used as a control, and chemical killing was performed by incubating fresh *Pb* SPZ with 10 µg/mL nucleic acid stain Hoechst (33342, Themofisher) for 30 minutes in a 37°C water bath. Directly after, dead SPZs were washed 5 times with RPMI 1640 glutamax and recounted before use.

Intravenous immunization in the tail vein was performed after warming of the mice under a heat lamp set at 35°C to dilate the veins. For every immunization, 25,000 live or dead *Pb* SPZs were inoculated in 200 µL RPMI 1640 glutamax. SGE and medium were used as controls. For the SGE control, an equal amount of SGE was injected in 200 µL RPMI 1640 glutamax and for the medium control, 200 µl RPMI 1640 glutamax. Second immunizations were given 7 days after the first. All injections were given between 1:00 PM and 3:00 PM.

### Determination of Parasite Liver Load After First and Second Immunization by Real-time *In Vivo* Imaging

After 44 hours post immunization, the parasitic liver load was determined through bioluminescent imaging. Imaging was performed using the IVIS Lumina II Imaging System (Perkin Elmer Life Sciences) [21] 12 minutes after a subcutaneous injection with D-luciferin dissolved in PBS (100 mg/kg; Caliper Life Sciences). Quantitative analysis of bioluminescence of whole bodies was performed by measuring the luminescence signal intensity using the region of interest settings of the Living Image® 4.4 software. Blood-stage breakthroughs were checked at 5 to 6 days post first immunization and second immunization. If any parasites were discovered in the blood, the mice were sacrificed and eliminated from the experiment. This happened to one mouse after the second immunization.

### Organ Harvesting and Processing

Two days or 7 days after the final immunizations, mice were anesthetized via intraperitoneal injection of 10% ketamine (Dechra Pharamceuticals) with 20 mg/mL xylazine (Alfasan). Blood was subsequently collected via retro-orbital puncture and put on ice. The blood was spun down at 4°C. Plasma was collected and stored at −80°C. The liver and lungs were perfused by slowly injecting 20 mL cold PBS via the heart, followed by dissection of the liver and lungs, and placed in sterile RPMI 1640 glutamax on ice.

Livers were processed via mincing with a blade and placed in a 50-mL tube with 20 mL RPMI 1640 glutamax containing 1 mg/mL Collagenase IV (Sigma-Aldrich) and 2,000 U/mL DNase I (Sigma-Aldrich) and incubated for 45 minutes at 37°C, mixing once during incubation. After incubation, tubes were placed on ice and poured through a 100-micron filter (BD) and washed with 20 mL PBS supplemented with 1% Fetal Calf Serum (FCS) and 2.5 mM ethylenediamine tetra-acetic acid (EDTA, Sigma-Aldrich). The tubes were spun down at 1,500 rpm for 10 minutes at 4°C and supernatants were gently taken off, after which PBS supplemented with 1% FCS and 2.5 mM EDTA was added to the pellets and tubes were spun down at 50 g for 3 minutes at 4°C. To separate the immune cells from the hepatocytes, the supernatant, which contains immune cells, was gently removed. The immune cells were spun down for 10 minutes at 1,600 rpm at 4°C. The supernatant was discarded, and 3 mL sterile PBS supplemented with 0.15M NH_4_Cl; 1mM KHCO_3_; 0.1 mM Na_2_EDTA was added for 2 minutes to lyse red blood cells, followed by the addition of 7 mL PBS supplemented with 1% FCS and 2.5 mM EDTA (Sigma-Aldrich). The tubes were spun down for 10 minutes at 1,600 rpm at 4°C. The supernatant was discarded, and the pellet was resuspended in 10 mL PBS supplemented with 0.5% bovine serum albumin (Fraction V, Roche) and 10 mM ETDA (Sigma-Aldrich) and spun down for 10 minutes at 1,200 rpm at 4°C. The supernatant was removed, and 35 µL CD45 MicroBeads (Miltenyi Biotec) were added and incubated for 15 minutes in the fridge. After incubation the left-over beads were washed off by adding 10 mL PBS supplemented with 0.5% bovine serum albumin (Fraction V, Roche) and 10 mM ETDA and spun down for 10 minutes at 1,600 rpm at 4°C. The pellets were resuspended in 5 mL PBS supplemented with 0.5% BSA (Fraction V, Roche) and 10 mM ETDA and run through a pre-wetted LS magnetic column according to protocol (Miltenyi Biotec). The columns were washed with RPMI 1640 glutamax, 5% FCS, 0.1% β-mercaptoethanol, 100U/mL penicillin, and 100 µg/mL streptomycin (culture medium) to collect the CD45^+^ cells. The tubes were spun down for 10 minutes at 1,600 rpm at 4°C, supernatants were taken off and resuspended in 5 mL washing medium, and viable cells were counted using trypan blue and a Bürker counting chamber.

To process lungs, the lungs were chopped into small pieces using a scalpel and transferred to a 15-mL tube. To digest the tissue 5 mL RPMI 1640 glutamax supplemented with 1 mg/mL Collagenase IV (Sigma-Aldrich), 2 µL/mL DNase (Sigma-Aldrich), and 2 µL/mL of 1M CaCL_2_ (Sigma-Aldrich) and incubated for 30 minutes at 37°C. After incubation, 10 mL cold RPMI 1640 glutamax with 5% FCS was added and poured through a 100-micron filter (BD). A 1 mL syringe was used to mash the digested tissue and washed with 20 mL cold RPMI 1640 glutamax with 5% FCS. The tubes were spun down for 10 minutes at 1,600 rpm at 4°C, and 2 mL sterile PBS supplemented with 0.15M NH_4_Cl; 1mM KHCO_3_; 0.1 mM Na_2_EDTA was added for 2 minutes to lyse the red blood cells. After 2 minutes 8 mL of washing medium was added, and tubes were spun down for 10 minutes at 1,600 rpm at 4°C. Pellets were resuspended in 5 mL of washing medium, and viable cells were counted using trypan blue and a Bürker counting chamber.

### Cell Stimulation and Analysis

A total of 200,000 cells per well were added. For specific restimulation, live *Plasmodium berghei* LA-GAP PbΔ*mei2* SPZ in a concentration of 25,000/mL (5,000 per 200,000 cells) in culture medium were added to each well and spun down for 4 minutes at 1,200 rpm at 4°C. For the specific restimulation, 0.1 µg/mL Phorbol 12-myristate 13-acetate (PMA) + 1 µg/mL ionomycin (Iono) (Sigma-Aldrich) in culture medium was added. To measure intracellular cytokine expression, 10 µg/mL Brefeldin A (Sigma-Aldrich) was directly added. All plates for flow-cytometry were incubated for 4 hours at 37°C with 5% CO_2_.

After 4 hours (flow cytometry measurement), the cells were transferred into a V-bottom plate and washed with cold PBS. Cells were stained with live/dead marker Zombie NIR (Thermo Fisher), fixed with eBioscience FOXP3/Transcription Factor Fixation/Permeabilization kit, and stained with different markers for 3 different panels: (1) Myeloid panel (Table 1), (2) T-cell panel (Table 2), or (3) Advanced T-cell panel (Table 3, implemented halfway through the experiments). To all panels, Fc-block (BD bioscience), True-Stain Monocyte Blocker (BioLegend), and Brilliant Violet buffer (Thermo Fisher) was added. The cells were measured by flow cytometry using Aurora 5 laser (Cytek Bioscience B.V., Amsterdam) and analyzed using Spectroflow (Cytek Bioscience B.V.), FlowJo version 10.8 (FlowJo LLC), and R-studio version 1.4.1717. For gating strategy see Supplementary Figure 1.

**Table 1.** Myeloid Panel

Target	Antibody clone	Fluorochrome
MHCII	2G9	BUV395
PDL1	B7-H1	BUV737
CD11c	N418	BV421
Siglec F	E50-2440	BV480
CD40	3–23	BV510
CCR7	4B12	BV605
XCR1	ZET	BV650
F4/80	T45-2342	BV711
CD45	30-f11	BV785
CD70	FR70	FITC
Ly6C	HK1.4	PerCP-Cy5.5
CD80	16-10A1	PE
CD64	X54-5/7.1	PE-Dazzle
B220	RA3-6B2	PE-Cy5
CD11b	M1/70	PE-Cy7
CD206	C068C2	APC
TIM4	F31-5G3	AF647
CD86	GL1	AF700
NK1.1	PK135	APC-Cy7

**Table 2.** T-cell Panel

Target	Antibody clone	Fluorochrome
CD3	17A2	BUV661
CD45	30-F11	BUV805
CXCR3	CXCR3-173	Super Bright 436
Ki67	SolA15	eFluor 506
γδ T-cells	GL3	BV605
CD4	GK1.5	BV650
PDL1	10F.9G2	BV711
PD1	29f.1A12	BV785
Perforin	eBio0mak	FITC
CD44	1M7	AF532
CD8	53-6.7	PerCP
Granzyme A	GrA-368.5	PerCP-ef710
CD137	4-1BB	PE
CD69	H1.2F3	PE-cf594
IFNγ	XMG1.2	PE-Cy5
KLRG1	2F1	PE-Cy5.5
TNFα	MPG-XT22	PE-Cy7
FOXP3	FjK-16s	APC
Granzyme B	NGZB	efluor 660
CD25	PC61.5	AF700

**Table 3.** Advanced T-cell Panel

Target	Antibody clone	Fluorochrome
MHCII	2G9	BUV395
CD25	PC 61.5	BUV563
CD3	17A2	BUV661
CD62L	Mel-14	BUV737
CD45	30-f11	BUV805
CXCR3	CXCR3-173	Super Bright 436
γδ T-cells	GL3	BV605
CD4	GK1.5	BV650
CD11c	N418	BV711
PD1	29f.1A12	BV785
CD40L	SA047C3	FITC
CD44	IM7	AF532
CD8	53-6.7	PerCP
CD64	X54-5/7.1	PerCP-efluor 710
CD69	H1.2F3	PE-cf594
IFNγ	XMG1.2	PE-Cy5
KLRG1	2F1	PE-Cy5.5
TNFα	MPG-XT22	PE-Cy7
CD86	GL1	AF700
NK1.1	PK135	APC-Cy7

## STATISTICAL ANALYSIS

Data were analyzed using Spectroflow (Cytek Bioscience B.V.), FlowJo version 10.8 (FlowJo LLC), R studio version 4.3.1, and Adobe illustrator 2023. Sample size estimation was performed with a power analysis using an alpha of 0.05 and a power of 90%. Statistical analyses were performed using one-way ANOVA with multiple comparisons with R studio version 4.3.1. Significance was defined as a p-value of less than 0.05. Data subjected to parametric statistical analyses had its normality confirmed beforehand.

## RESULTS

### Distribution of Immune Cells in Liver and Lungs

To evaluate the potency of LA-GAP whole SPZ immunization, we analyzed the load of liver-stage parasites by bioluminescence. We confirmed that parasite liver loads were negative after the second immunization, suggesting full protection (Supplementary Figure 2). First, we investigated the overall kinetics of immune cell distribution in the liver and lungs 2 and 7 days after the last immunization. At the early timepoint we specifically looked at the innate cell compartments and found no differences in total number of myeloid cells (monocytes, dendritic cells, macrophages, neutrophils, and Kupffer cells in the liver) or B-cells in both liver and lungs after SPZ immunization compared with salivary gland extract (SGE, data not shown).

**Figure 2.**
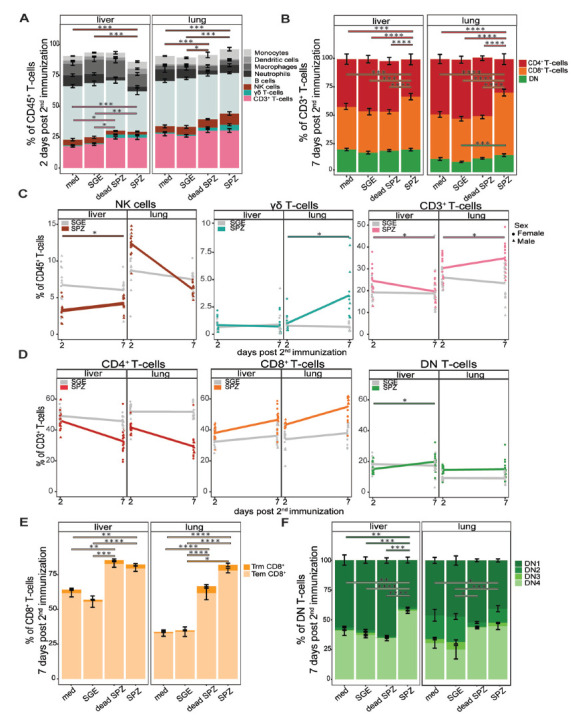
**Distribution of different cell types.** (A) Percentage (gated out of CD45^+^ live cells) of monocytes (light grey), dendritic cells (grey), macrophages (dark grey), neutrophils (black), B-cells (blue grey), NK cells (brown), γδ T cells (turquoise), CD3^+^ T cells (pink), in liver and lungs after immunization with medium (med), salivary gland extract (SGE), dead sporozoites (SPZ), or live SPZ, 2 days post second immunization. (B) Distribution of CD4^+^ T cells (red), CD8^+^ T cells (orange), and DN T-cells (green), in liver and lungs after immunization with medium, SGE, dead SPZ, or SPZ, 7 days post second immunization. (C) Changes in distribution of NK cells, γδ T cells, or CD3^+^ T cells over time between days 2 to 7 post second immunization. SGE-injected mice in grey, SPZ-injected mice in brown for NK cells, turquoise for γδ T cells, or pink for CD3^+^ T cells. (D) Changes in distribution of CD4^+^ T cells, CD8^+^ T cells, or DN T cells over time between days 2 to 7 post second immunization. SGE-injected mice in grey, SPZ-injected mice in red for CD4^+^ T cells, orange for CD8^+^ T cells, or green for DN T cells. (E) Number of Tem (light orange) and Trm (orange) CD8^+^ T cells in liver and lungs after immunization with medium, SGE, dead SPZ, or SPZ, 7 days post second immunization. (F) Number of DN1, DN2, DN3, and DN4 cells in different shades of green in liver and lungs after immunization with medium, SGE, dead SPZ, or SPZ, 7 days post second immunization. Med n=14, SGE n=18, dead SPZ n=10, SPZ n=18, divided over 2 (dead SPZ) or 3 (med, SGE, SPZ) experiments. Statistical significance between groups was assessed by one-way ANOVA with multiple comparisons. *P<0.05, **P<0.005, ***P<0.0005, and ****P<0.0001.

In the liver, we observed an early (2-day) increase of CD3^+^ T cells (*P*=0.003, Figure 2A, C); whereas, in the lungs, we observed an early (2-day) and late (7-day) increase of CD3^+^ T-cells after immunization of SPZs compared to SGE (*P*=0.003, Figure 2C). We subsequently gated the CD3^+^ T-cell population on expression of CD4 and CD8 and found the T-cell increase was mainly attributed to an increased percentage of CD8^+^ T cells (*P*<0.0001, *P*<0.0001, SPZ vs SGE for liver and lungs) with a reciprocal decrease in CD4^+^ T cells (*P*<0.0001, *P*<0.0001, SPZ vs SGE for liver and lungs, respectively, Figure 2B). Interestingly, after immunization with dead SPZs we observed similar trends of immune cell recruitment, although the magnitude of CD8^+^ T-cell recruitment seems to be lower in both organs as compared to live SPZ immunization (Figure 2B).

The dynamics of the CD3^+^ T cells showed an overall important increase from day 2 to day 7 after immunization (mean increase 4.5%) in the lungs, whereas the reciprocal was true in the liver with overall CD3^+^ cells decreased slightly over time (Figure 2C). Also, the total number of CD4^-^ CD8^-^ (double negative, DN) T cells (*P*=0.0003, Figure 2B) and Trm CD8^+^ T cells (CD44^hi^ CD62L^-^CD69^+^) (*P*=0.04, Figure 2E) increased from day 2 to 7 days after immunization in the lungs but not in the livers. Similarly, γδ T cells in the lungs showed a large increase of 2.5% over time but remained stable in the liver (Figure 2C), indicating that the total number of both CD3^+^ and γδ T cells expands over time in the lungs but not in the liver. Moreover, we observed a significant increase in total number of natural killer (NK) cells (*P*=0.0009, Figure 2A) 2 days after immunization with SPZs in the lungs but not the livers. The early increased percentage of NK cells in the lungs was of a short duration as they decreased already at day 7 (mean 12.4% to 6.2% over time, Figure 2C). Finally, we found over time a trend for increased CD8^+^ T-cell percentage in both organs (Figure 2D).

Within the CD8^+^ T-cell population, mainly Tem CD8^+^ T cells (CD44^hi^ CD62L^-^ CD69^-^) were increased (*P*<0.0001, *P*<0.0001 SPZ vs SGE for liver and lungs, respectively, Figure 2E). The number of Trm CD8^+^ T cells (CD44^hi^ CD62L^-^ CD69^+^) was significantly increased in the lungs only (*P*=0.04, Figure 2E). Overall, the DN T cells showed a complex dynamic, with a stable increase in the lungs only (Figure 2D). Expression of CD44 and CD25 on DN T cells significantly decreased in the liver (*P*=0.0001) and showed a decreased trend of expression in the lungs after SPZ immunization. This resulted in an increase of DN4 cells (*P*<0.0001, *P*=0.0003 SPZ vs SGE for liver and lungs, respectively), which are known for differentiation into γδ T cells, CD4^+^ or CD8^+^ T cells (Figure 2F).

Collectively, changes in immune cell distribution after SPZ immunization, notably in the NK cell population, the overall CD3^+^ T-cell population, γδ T-cell population, and CD8^+^ T-cell population, in particular the Trm CD8^+^ T-cells, were more pronounced in the lungs than the liver. In general, the lungs thus seem to recruit a more diverse pallet of different cell types, and for a longer duration after SPZ immunization as compared with the liver.

### Cellular Activation

**Figure 3.**
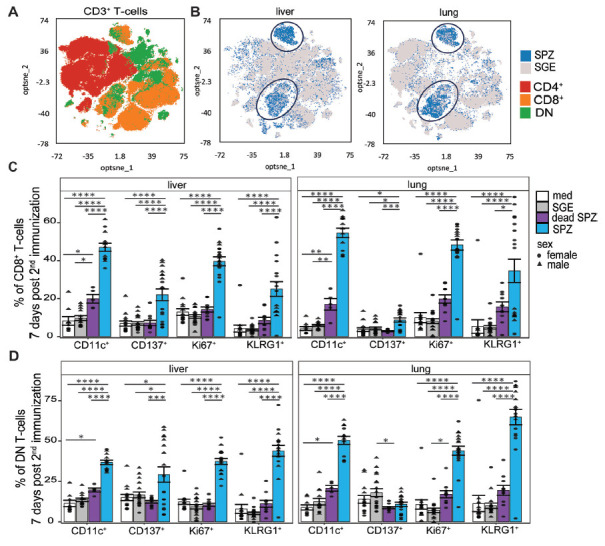
**Cellular activation.** (A) Optimized t-Distributed Stochastic Neighbor Embedding with CD8^+^ T cells in orange, CD4^+^ T-cells in red, and DN T-cells in green. (B) Optimized t-Distributed Stochastic Neighbor Embedding T-cell overlay of SPZ-injected (blue) or SGE-injected (grey) mice. (C) CD11c^+^, CD137^+^, Ki67^+^, and KLRG1^+^ CD8^+^ T cells after immunization with medium (white), SGE (grey), dead SPZ (purple), or SPZ (blue). (D) CD11c^+^, CD137^+^, Ki67^+^, and KLRG1^+^ DN T cells after immunization with medium (white), SGE (grey), dead SPZ (purple), or SPZ (blue) 7 days post second immunization. Male mice showed by triangle and female mice showed by circle symbol, restimulated with SPZs. CD137, Ki67, and KLRG1: med n=14, SGE n=18, dead SPZ n=10, SPZ n=18, divided over 2 (dead SPZ) or 3 (med, SGE, SPZ) experiments. CD11c: med n=9, SGE n=13, dead SPZ n=9, SPZ n=13, divided over 2 experiments. Statistical significance between groups was assessed by one-way ANOVA with multiple comparisons. *P<0.05, **P<0.005, ***P<0.0005, and ****P<0.0001.

Having shown dynamic changes in mainly T cells in both organs, we next investigated the activation status of these cells at 7 days post second immunization following *ex vivo* restimulation with SPZs. An optimized t-Distributed Stochastic Neighbor Embedding (opt-SNE) analysis based on different T-cell markers revealed activation of 2 cell clusters after SPZ immunization, which were not activated after SGE immunization. We found that these clusters consisted of CD8^+^ T cells and DN T cells (Figure 3A+B). Activation of the CD8^+^ T cells was characterized by an increase in CD11c (*P*<0.0001, *P*<0.0001), Ki67 (*P*<0.0001, *P*<0.0001), and KLRG1 (*P*<0.0001, *P*<0.0001) in both liver and lungs after SPZ immunization compared with SGE (Figure 3C, Supplementary Figure 3A+B). Impressively, we observed that almost half of the CD8^+^ T cells express CD11c and/or Ki67 in both liver and lungs after SPZ immunization. In the DN T cells, we observed a similar pattern of activation markers for both organs after SPZ immunization, with increases in CD11c (*P*<0.0001, *P*<0.0001), Ki67 (*P*<0.0001, *P*<0.0001), and KLRG1 (*P*<0.0001, *P*<0.0001) (Figure 3D, Supplementary Figure 3C+D). Over time we observed in both organs an increased percentage of Ki67^+^ CD8^+^ T cells and Ki67^+^ DN T cells, while the percentage of KLRG1^+^ CD8^+^ T cells, KLRG1^+^ DN T cells, CD11c^+^ CD8^+^ T cells, and CD11c^+^ DN T cells stayed stable (Supplementary Figure 3E+F). We found an increase in CD137^+^ DN^+^ T cells for the liver only (*P*=0.02) and not for the lungs. On the contrary, the lungs showed an increased expression of proliferation marker Ki67 and a significant increase in proliferation marker KLRG1 on γδ T cells (*P*=0.0004, Supplementary Figure 4A). Interestingly, we hardly observed any activation of immune cells after immunization with dead SPZs in both liver and lungs (Figure 3C+D, Supplementary Figure 3A, B, C, D, Supplementary Figure 5A).

As for the innate compartments 2 days post second immunization, both organs showed an increased trend in expression of KLRG1 on NK cells after SPZ immunization (Supplementary Figure 4B). In the liver, macrophages showed an upregulation of both activation and regulatory markers (CD80 *P*=0.0006 and PDL1 *P*=0.007, Supplementary Figure 5B), whereas in the lungs, no activation of macrophages was found but only increases in the regulatory marker PDL-1 (Supplementary Figure 5A). These regulatory macrophages were further phenotyped as interstitial macrophages (Supplementary Figure 6C). In the liver we found an increased trend in expression on monocytes (CD80 and CD86, Supplementary Figure 6A), which was absent in the lungs.

In conclusion, activated cells were mainly found within the CD8^+^ T cell and DN T cell compartments and after immunization with live SPZs. Here, there were only slight phenotypic differences between lung and liver. However, the phenotype of macrophages and monocytes was different between liver and lungs, with activation markers on both cells only found in the liver.

### T-cell Killing Pathway

**Figure 4.**
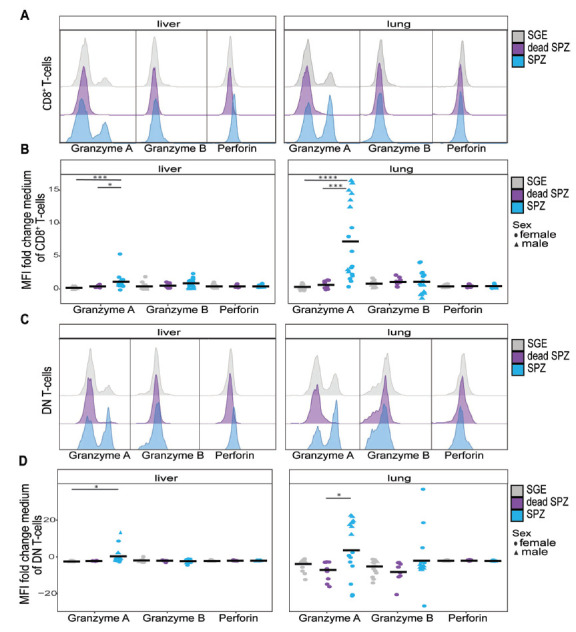
**T-cell functionality.** (A) Histogram of Granzyme A, Granzyme B, or perforin expression on CD8^+^ T cells after SGE (grey), dead SPZ (purple), or SPZ (blue) immunization. (B) Mean expression of Granzyme A, Granzyme B, or perforin on CD8^+^ T cells after SGE (grey), dead SPZ (purple), or SPZ (blue) immunization, fold change to medium. (C) Histogram of Granzyme A, Granzyme B, or perforin expression on DN T cells after SGE (grey), dead SPZ (purple), or SPZ (blue) immunization. (D) Mean expression of Granzyme A, Granzyme B, or perforin on DN T cells after SGE (grey), dead SPZ (purple), or SPZ (blue) immunization, fold change to medium. All data from 7 days post second immunization. Male mice showed by triangle and female mice showed by circle symbol, cells restimulated with SPZ. Med n=14, SGE n=18, dead SPZ n=10, SPZ n=18, divided over 2 (dead SPZ) or 3 (med, SGE, SPZ) experiments. Statistical significance between groups was assessed by one-way ANOVA with multiple comparisons. *P<0.05, **P<0.005, ***P<0.0005, and ****P<0.0001.

Having shown an increase in percentage of activated CD8^+^ T cells and DN T cells in the liver and lungs, we next investigated the functionality of these cells by measuring Granzyme A, Granzyme B, and perforin expression 7 days after the last immunization after *ex vivo* restimulation with SPZ. Interestingly, we observed that CD8^+^ T cells in both organs displayed an increased expression of Granzyme A but not Granzyme B or perforin when injected with SPZs compared with SGE (*P*=0.0002, *P*<0.0001, Figure 4A+B+C+D). Again, this observation was specific for live SPZ immunization only, as Granzyme A expression was not increased after dead SPZ immunization (Figure 4B+D).

When looking into organ-specific differences, we found the magnitude of expression of Granzyme A was roughly 4-fold higher in lungs as compared to liver. For DN T cells, on the contrary, we observed a significant increase of Granzyme A expression in the liver (*P*=0.015) and a lower trend of increased expression in the lungs after SPZ immunization compared with SGE immunization (Figure 4C+D). Interestingly, here we observed some sex-specific differences in immune functionality where, for both the CD8^+^ T cells and DN T cells in the lungs, a higher expression of Granzyme A appeared in male mice than in female mice (Figure 4B+D).

Collectively, functionality of CD8^+^ T cells and DN T cells seems to be mediated by Granzyme A after SPZ immunization in the lungs.

### T-cell Cytokine Expression

**Figure 5.**
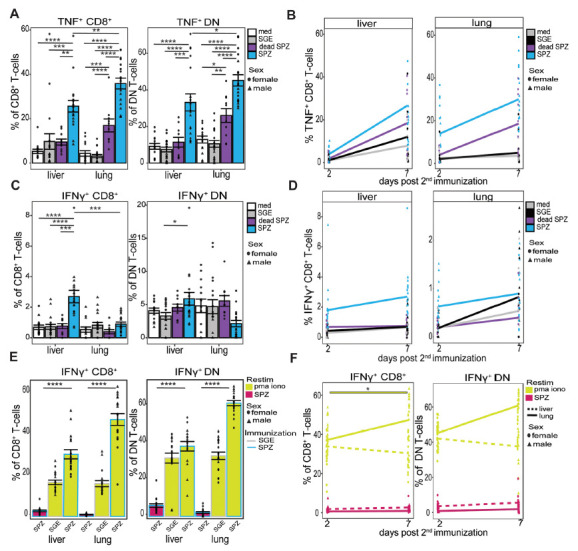
**T-cell cytokine functionality.** (A) Percentage of TNF^+^ CD8^+^ T cells and TNF^+^ DN T cells after medium (white), SGE (grey), dead SPZ (purple), or SPZ (blue) 7 days post second immunization after SPZ restimulation. (B) Percentage of TNF^+^ CD8^+^ T cells over time, from 2 to 7 days post second immunization after SPZ restimulation. (C) Percentage of IFNγ^+^ CD8^+^ T cells and IFNγ^+^ DN T cells after medium (white), SGE (grey), dead SPZ (purple), or SPZ (blue) 7 days post second immunization after SPZ restimulation. (D) Percentage of IFNγ^+^ CD8^+^ T cells over time, from 2 to 7 days post second immunization after SPZ restimulation. (E) Percentage IFNγ^+^ CD8^+^ T cells and IFNγ^+^ DN T cells of SPZ or SGE injected mice after SPZ (pink) or PMA/Iono (yellow) restimulation, 7 days post second immunization. (F) Percentage IFNγ^+^ CD8^+^ T cells and IFNγ^+^ DN T cells over time from 2 to 7 days post second immunization, following SPZ (pink) or PMA/Iono (yellow) restimulation. Interrupted line for liver and smooth line for lungs. Med n=14, SGE n=18, dead SPZ n=10, SPZ n=18, divided over 2 (dead SPZ) or 3 (med, SGE, SPZ) experiments. Male mice showed by triangle and female mice showed by circle symbol. Statistical significance between groups was assessed by one-way ANOVA with multiple comparisons. *P<0.05, **P<0.005, ***P<0.0005 and ****: P<0.0001.

Finally, we investigated the cytokine expression patterns of these CD8^+^ T cells and DN T cells in the liver and lungs 7 days post second immunization and after *ex vivo* restimulation with SPZs by measuring intracellular TNF and IFNγ by flow cytometry. Here, we observed a significant increase of TNF expression in CD8^+^ T cells (*P*=0.0002) and DN T cells (*P*<0.0001) in the liver and CD8^+^ T cells (*P*<0.0001) and DN T cells (*P*<0.0001) in the lungs (Figure 5A). This was specific to live SPZ immunization, as the increased expression of TNF after dead SPZ immunization was modest and significantly lower compared with live SPZ (liver; *P*=0.001, lung; *P*<0.0001). Increases were more pronounced in the lungs as compared with the liver (TNF^+^ CD8^+^ T cells *P*=0.007 and TNF^+^ DN T-cells *P*=0.04, Figure 5A). These differences start already at the early 2-day timepoint at which the lungs show a 10.2% increase in TNF^+^ CD8^+^ T cells and the liver shows a more modest increase of 2.6% (Figure 5B, Supplementary Figure 5A). Moreover, γδ T cells showed an increased expression of TNF only in the lungs after SPZ immunization compared with SGE (*P*=0.007) but not in the liver (Supplementary Figure 5E).

For IFNγ, we observed a different pattern of expression, as IFNγ^+^ CD8^+^ T cells increase only in the liver (2.7% increase SPZ vs SGE) and not in the lungs (Figure 5C). This increase resulted in a significant difference in IFNγ^+^ CD8^+^ T cells between the liver and lungs (*P*=0.0007, Figure 5C). However, *ex vivo* restimulation with PMA/Iono instead of SPZs, which says something about the functionality of the cells instead of specificity, showed an increase of IFNγ^+^ CD8^+^ T cells (*P*<0.0001) and IFNγ^+^ DN T cells (*P*<0.0001) in the lung, which was 17% (CD8^+^ T cells) and 23% (DN T cells) higher compared with the liver (Figure 5E+F).

Also CD4^+^ T-cells expressed IFNγ to a higher extent in the liver as compared with the lungs after SPZ immunization with SPZ restimulation. Similarly to the CD8^+^ T-cells, these cells could be readily activated with a 2-fold increase in IFNγ expression CD4^+^ T-cells and a 7-fold increase in γδ T-cells in the lungs after *ex vivo* restimulation with PMA/Iono compared with SPZ, which was not observed in the liver (Supplementary Figure 5D+F).

Overall, we demonstrated that mainly CD8^+^ T-cells and DN T cells in the lungs are increased in number and activation after SPZ immunization. These lung T cells express Granzyme A and TNF after SPZ restimulation but less IFNγ. After aspecific PMA/Iono restimulation, however, they also readily produce IFNγ.

## DISCUSSION

Here, we compared the immune response to LA-GAP immunization in the liver and lungs in a rodent malaria model. Surprisingly, we found that responses in the lungs were overall more pronounced than in liver, particularly in the CD8^+^ DN, and γδ T-cell compartments, and lung cells displayed an increased expression of pro-inflammatory activation markers and cytokines. Most immune changes were specific to immunization with live SPZ and could not be found after immunization with dead SPZ. These results thus suggest that the lungs may be a key organ in mediating protection after LA-GAP immunization.

SPZ make use of the host circulatory system to travel to the liver. Inherent to the anatomy of the circulatory system, SPZ must pass the lungs before being able to reach the target organ. Because of the narrow diameter of lung capillaries (3–5 µm in mice [22]), SPZ (10–15 µm in length and 1.5 µm in width [23]) must actively navigate to pass this organ. This short interaction of the SPZ with its surrounding tissue provides the host with an opportunity to harness innate cells such as macrophages to capture SPZ and activate the adaptive immune system [24–26]. However, the overall impact of the interaction and the ensuing immune response has not been studied before.

For other hematogenous parasites like *Schistosoma* and *Trypanosoma* and parasites that utilize the bloodstream such as *Toxoplasma* and *Leishmania,* it has been known that they can activate CD8^+^ T cells and kill parasite infected cells in the lungs [27–30]. Moreover, from pulmonary viral and bacterial infections, it is known that lung CD8^+^ T cells can upregulate multiple activation markers such as IFNγ and TNF in different murine models and that these activated cells are critical for mediating pathogen clearance [31, 32]. Even though the route of entering the lungs might differ per pathogen, similarities in the expression profile of IFNγ and TNF on CD8^+^ T cells makes us hypothesize that the immune cells in the lungs play a comparable role in mediating SPZ clearance after immunization of LA-GAP SPZ.

Also, for *Plasmodium*, CD8^+^ T cells were shown to mediate critical protection in rodent and non-human primate studies. Using radiation-attenuated SPZ (RAS) immunization models, DCs in the spleen, liver, and liver-draining lymph nodes were shown to present antigens, likely via antigen cross-presentation, to CD8^+^ T cells [33–35]. Depletion of CD8^+^ T cells consequently abrogated the immunity induced by RAS immunization, proving they play a key role in mediating protection in the liver [36]. Moreover, liver Trm CD8^+^ T cells were able to directly kill infected hepatocytes through secretion of perforin and Granzyme B [9, 19] likely with a complementary function of Granzyme A in early responses [37]. In the current study, we find a similar phenotype of CD8^+^ T cells in the lungs, again supporting the hypothesis that these cells may also be able to mediate protection. The regulatory macrophage phenotype, which we found earlier in the lungs, has previously been described after SPZ uptake [38], leading us to believe that lung macrophages are able to take up SPZ and may play a role in T-cell priming after LA-GAP inoculation.

However, it remains unclear whether these CD8^+^ T cells, once primed in the lungs, are capable of directly killing SPZs within the lungs, thus reducing the number of SPZs that reach the liver, or if they migrate to the liver to specifically target infected hepatocytes. However, judging by their phenotype, the Tem cells we identified in the lungs, unlike Trm, retain the ability to migrate to other organs. In addition, we observed a stark increase of CD11c^+^ CD8^+^ T cells, which co-express Ki67 and KLRG1, in the lungs. It has been proven that cells with the same phenotype in the liver can inhibit liver-stage parasite development *in vitro* [39]. This suggests that T cells may initially be primed in the lungs before migrating to the liver, where they could potentially eliminate infected hepatocytes. The impact of these findings on CD8^+^-mediated killing of malaria-infected hepatocytes thus depends on their capability to recirculate.

Despite considerable differences between humans and rodents, we argue that our findings are nonetheless relevant to human LA-GAP programs. The pulmonary capillary diameters of mice (3–5 µm [22]) and humans (1–7.5 µm, mean 3.7 µm) are comparable [40], meaning that human immune cells can similarly encounter SPZs in the lungs. Moreover, we observed a comparable T-cell increase and phenotype of CD69 expressing CD8^+^ Trm T cells in mice lungs and livers as has been described in human livers of ChAd63 MVA ME-TRAP vaccinated volunteers associated with a reduced risk of developing malaria [41]. In a recent human clinical trial with mosquito bite-administered LA-GAP, we showed high-level protection could be achieved with just a single immunisation [42]. How a potential future change in the route of administration from mosquito bite to direct venous inoculation, with higher numbers of LA-GAP passing the lung, may potentially affect downstream protection remains to be investigated.

In conclusion, we compared organ-specific immune responses in lungs and liver after LA-GAP immunization and found impressive activation of CD8^+^ and DN T cells, particularly in the lungs. These observations suggest a pivotal role for the lungs as a priming immune organ and warrant further investigation to further our understanding of the full potential of LA-GAP immunization as a powerful tool to combat malaria.

## SUPPLEMENTARY DATA

Supplementary materials are available at the *Pathogens and Immunity* website. Supplementary data may be provided by the authors to benefit the reader. Supplementary data are not copyedited and are the sole responsibility of the authors. Questions or comments related to supplementary materials should be addressed to the corresponding author.
