# Supplementary material for: Early Activation of Lung CD8+ T Cells After Immunization with Live Plasmodium berghei Malaria Sporozoites
Source: Pathog Immun. 2025 Mar 4;10(2):46–68. doi: 10.20411/pai.v10i2.794 (PMC11888604; doi:10.20411/pai.v10i2.794)
Supplement: Supplementary Figures [file pai-10-046-s01.pdf]

PATHOGENS AND IMMUNITY
IN ASSOCIATION WITH CASE WESTERN RESERVE UNIVERSITY AND THE RICHARD J. FASENMYER FOUNDATION

Supplementary Figures

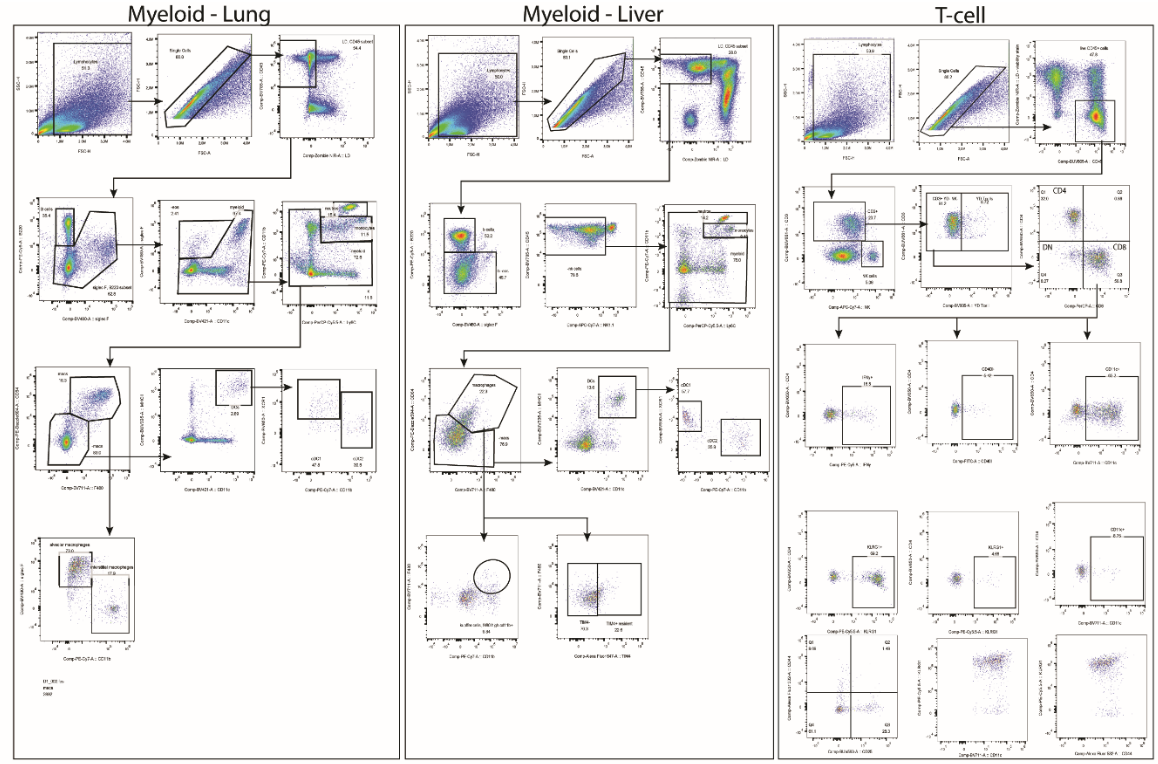

Supplementary Figure 1. Gating strategy. Gating strategy used to gate out liver and lung myeloid cells. Same gating strategy is used for the different experiments. All gating was performed in FlowJo.

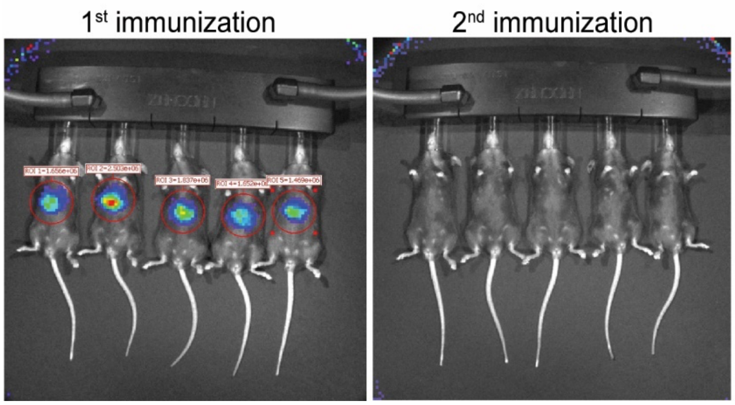

Supplementary Figure 2. liver load. Measurement of parasitic liver load by the expression of luciferase 44 hours after 1st immunization (left panel) and after 2nd immunization (right panel) with LA-GAP SPZ.

van Schuijlenburg R, Naar CM, van der Wees S, Chevalley-Maurel SC, Duszenko N, de Bes-Roeleveld HM, Iliopoulou E, Houlder EL, Geurten FJA, Baalbergen E, Roestenberg M, Franke-Fayard B. Early Activation of Lung CD8$^+$ T cells After Immunization with Live *Plasmodium berghei* Malaria Sporozoites. *Pathogens and Immunity*. 2025;10(2):46-68. doi: 10.20411/pai.v10i2.794

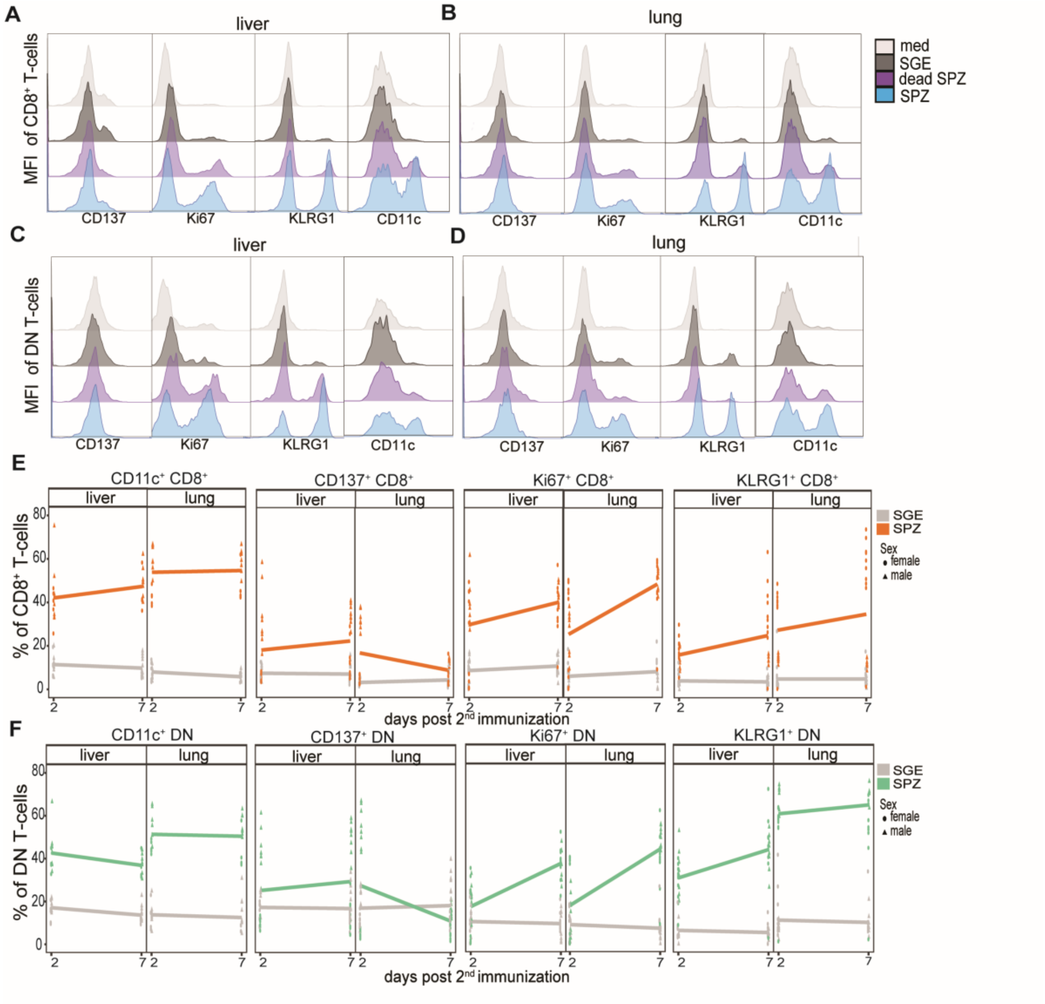

Supplementary Figure 3. Activation CD8$^+$ T-cells and DN T-cells histograms and expression over time. **(A)** Histogram of Medium Fluorescence intensity (MFI) of different markers on CD8$^+$ T-cells in the liver. **(B)** Histogram of MFI of different markers on CD8$^+$ T-cells in the lungs. (**C**) Histogram of MFI of different markers on DN T-cells in the liver. (**D**) Histogram of MFI of different markers on DN T-cells in the lungs. Med in grey, SGE in grey, dead SPZ in purple, SPZ in blue. (E) Percentage of different activation markers on CD8$^+$ T-cells over time (from 2 to 7 days after last immunization) in liver and lungs. SGE injected mice in grey, SPZ injected mice in orange. (**F**) Percentage of different activation markers on DN T-cells over time (from 2 to 7 days after last immunization) in liver and lungs. SGE injected mice in grey, SPZ injected mice in green. Med n=14, SGE n=18, dead SPZ n=10, SPZ n=18, divided over 2 (dead SPZ) or 3 (med, SGE, SPZ) experiments. Male mice in triangles and female mice in circles.

van Schuijlenburg R, Naar CM, van der Wees S, Chevalley-Maurel SC, Duszenko N, de Bes-Roeleveld HM, Iliopoulou E, Houlder EL, Geurten FJA, Baalbergen E, Roestenberg M, Franke-Fayard B. Early Activation of Lung CD8$^+$ T cells After Immunization with Live *Plasmodium berghei* Malaria Sporozoites. *Pathogens and Immunity*. 2025;10(2):46-68. doi: 10.20411/pai.v10i2.794

PATHOGENS AND IMMUNITY
IN ASSOCIATION WITH CASE WESTERN RESERVE UNIVERSITY AND THE RICHARD J. FASENMYER FOUNDATION

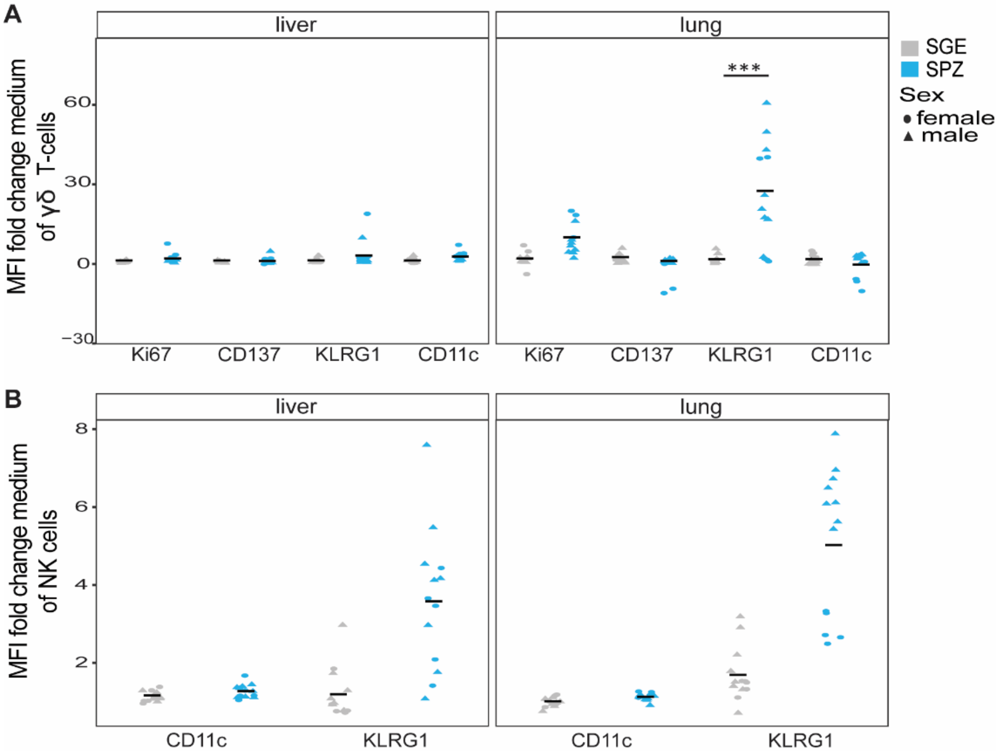

Supplementary Figure 4. Cellular activation γδ T-cells and NK-cells. Medium Fluorescence intensity (MFI) of different markers used to calculate fold change compared with medium (med). For the med group, mean of all mice per experiment was used. (**A**) Expression of different markers on γδ T-cells in the liver and lungs. (**B**) Expression of different markers on NK cells in the liver and lungs. In grey salivary gland extract (SGE) injected mice and in blue sporozoite (SPZ) injected mice. Med n=14, SGE n=18, dead SPZ n=10, SPZ n=18, divided over 2 (dead SPZ) or 3 (med, SGE, SPZ) experiments. Male mice in triangles and female mice in circles. Statistical significance between groups was assessed by one-way ANOVA with multiple comparisons. *: $P<0.05$, **: $P<0.005$, ***: $P<0.0005$ and ****: $P<0.0001$.

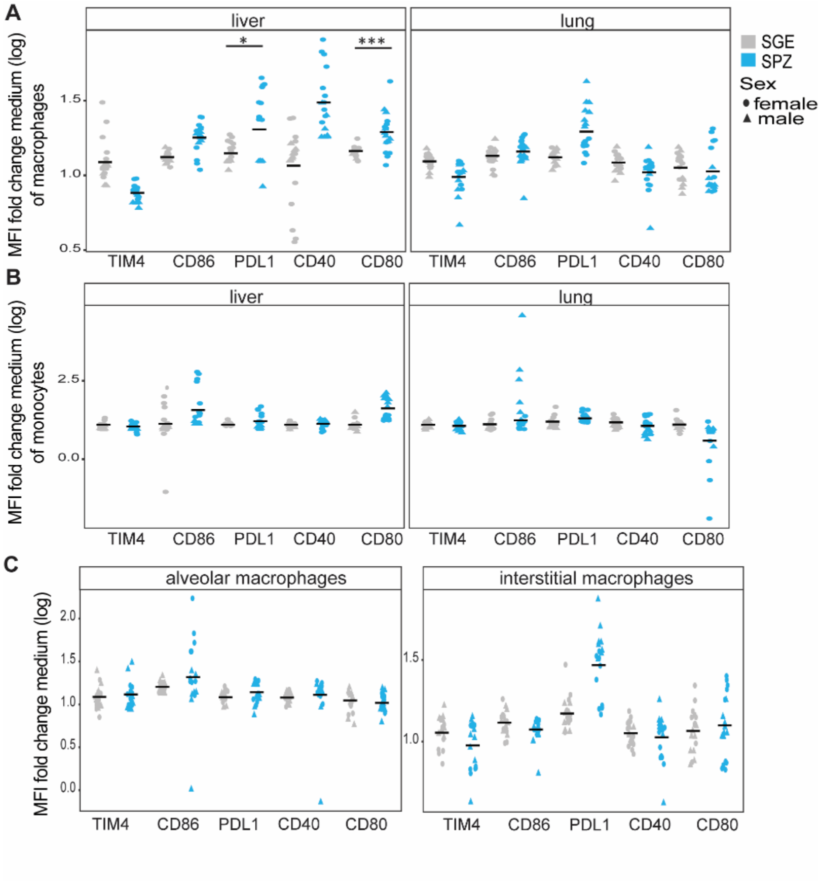

Supplementary Figure 5. Cellular activation myeloid and B-cells. Medium Fluorescence intensity (MFI) of different markers used to calculate fold change compared with medium (med). For the med group, mean of all mice per experiment was used. (**A**) Expression of different markers on macrophages in the liver and lungs. (**B**) Expression of different markers on monocytes in the liver and lungs. (**C**) Expression of different markers on alveolar and interstitial macrophages in the lungs. In grey salivary gland extract (SGE) injected mice and in blue sporozoite (SPZ) injected mice. Med n=14, SGE n=18, dead SPZ n=10, SPZ n=18, divided over 2 (dead SPZ) or 3 (med, SGE, SPZ) experiments. Male mice in triangles and female mice in circles. Statistical significance between groups was assessed by one-way ANOVA with multiple comparisons. *: *P*<0.05, **: *P*<0.005, ***: *P*<0.0005 and ****: *P*<0.0001.

van Schuijlenburg R, Naar CM, van der Wees S, Chevalley-Maurel SC, Duszenko N, de Bes-Roeleveld HM, Iliopoulou E, Houlder EL, Geurten FJA, Baalbergen E, Roestenberg M, Franke-Fayard B. Early Activation of Lung CD8^{+} T cells After Immunization with Live *Plasmodium berghei* Malaria Sporozoites. *Pathogens and Immunity*. 2025;10(2):46-68. doi: 10.20411/pai.v10i2.794

PATHOGENS AND IMMUNITY
In Association with Case Western Reserve University and The Richard J. Fasenmyer Foundation

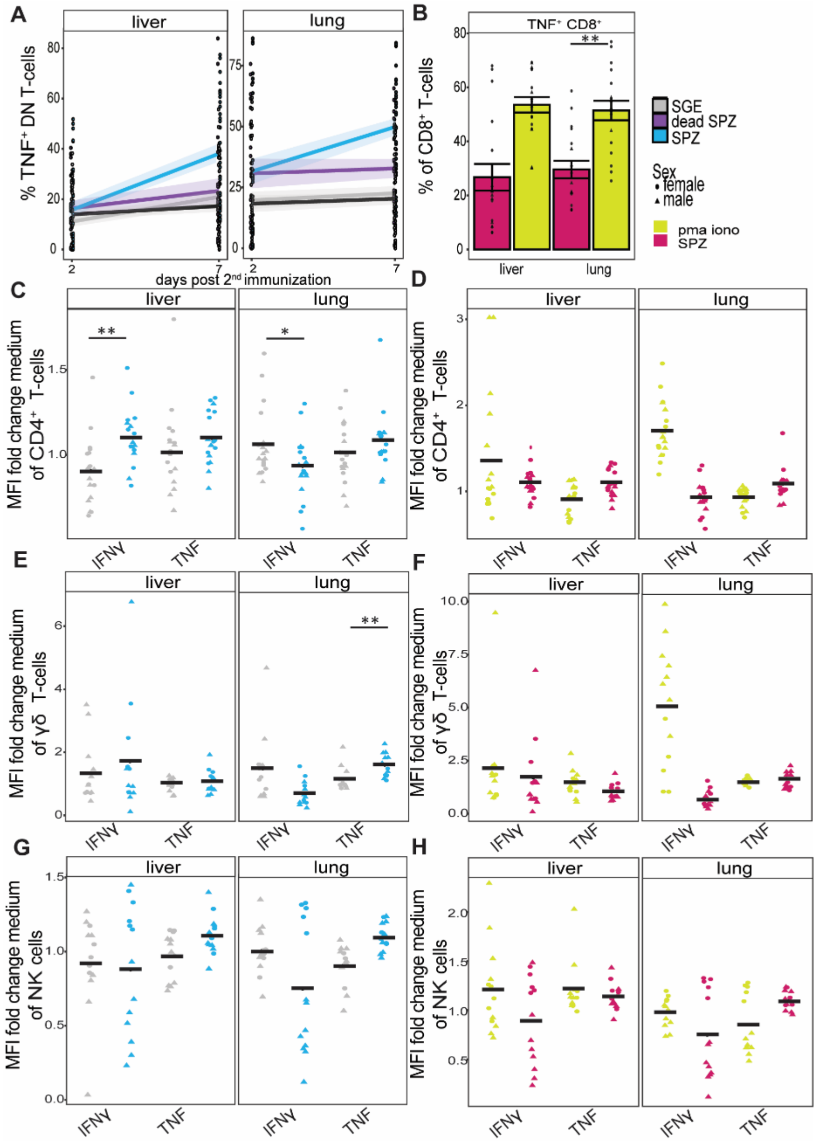

Supplementary Figure 6. Cytokine expression. (A) Percentage of TNF+ DN T-cells over time (time (from 2 to 7 days after last immunization) in liver and lungs. Med in grey, SGE in grey, dead SPZ in purple, SPZ in blue. **(B)** Percentage of TNF+CD8+ T-cells after PMA/Iono restimulation (yellow) or SPZ restimulation (pink) at 7 days after SPZ immunization in liver and lungs. (**C)** Fold change compared with medium expression of IFNγ and TNF on CD4+ T-cells in the liver and lungs after SGE (grey) or SPZ (blue) immunization. (**D)** Fold change expression of IFNγ and TNF on CD4+ T-cells after PMA/Iono (yellow) or SPZ (pink) restimulation in the liver and lungs compared with medium. **(E)** Fold change expression of IFNγ and TNF on γδ T-cells in the liver and lungs after SGE (grey) or SPZ (blue) immunization compared with medium. (**F)** Fold change expression of IFNγ and TNF on γδ T-cells after PMA/Iono (yellow) or SPZ (pink) restimulation in the liver and lungs compared with medium. (**G)** Fold change expression of IFNγ and TNF on NK cells in the liver and lungs after SGE (grey) or SPZ (blue) immunization compared with medium. (**H)** Fold change expression of IFNγ and TNF on NK cells after PMA/Iono (yellow) or SPZ (pink) restimulation in the liver and lungs compared with medium. Medium Fluorescence intensity (MFI) of different markers used to calculate fold change compared with medium (med). For the med group, mean of all mice per experiment were used. Med n=14, SGE n=18, dead SPZ n=10, SPZ n=18, divided over 2 (dead SPZ) or 3 (med, SGE, SPZ) experiments. Male mice in triangles and female mice in circles. Statistical significance between groups was assessed by one-way ANOVA with multiple comparisons. *: *P*<0.05, **: *P*<0.005, ***: *P*<0.0005 and ****: *P*<0.0001.

van Schuijlenburg R, Naar CM, van der Wees S, Chevalley-Maurel SC, Duszenko N, de Bes-Roeleveld HM, Iliopoulou E, Houlder EL, Geurten FJA, Baalbergen E, Roestenberg M, Franke-Fayard B. Early Activation of Lung CD8+ T cells After Immunization with Live *Plasmodium berghei* Malaria Sporozoites. *Pathogens and Immunity*. 2025;10(2):46-68. doi: 10.20411/pai.v10i2.794
